# Electroacupuncture for Women with Chronic Severe Functional Constipation: Subgroup Analysis of a Randomized Controlled Trial

**DOI:** 10.1155/2019/7491281

**Published:** 2019-01-13

**Authors:** Jing Zhou, Yan Liu, Kehua Zhou, Baoyan Liu, Tongsheng Su, Weiming Wang, Zhishun Liu

**Affiliations:** ^1^Department of Acupuncture, Guang'anmen Hospital, China Academy of Chinese Medical Sciences, Beijing, China; ^2^China Academy of Chinese Medical Sciences, Beijing, China; ^3^Institute of Basic Research in Clinical Medicine, China Academy of Chinese Medical Sciences, Beijing, China; ^4^Catholic Health System Internal Medicine Training Program, University at Buffalo, Buffalo, NY, USA; ^5^Shaanxi Province Hospital of Traditional Chinese Medicine, Xi'an, China

## Abstract

**Background:**

Acupuncture has been found to be effective for treating chronic constipation.

**Objective:**

The objective of this exploratory study was to evaluate the efficacy of electroacupuncture (EA) in the subgroup of women with chronic severe functional constipation.

**Methods:**

This is a subgroup analysis of the multicenter, randomized, sham-acupuncture (SA) controlled trial. The efficacy of 822 (76%) female patients of the 1075 randomized patients with chronic severe functional constipation was evaluated. Patients were randomly assigned to receive 28 sessions of EA or SA over 8 weeks with 12 weeks' follow-up. This study focused on sustained complete spontaneous bowel movements (CSBMs) responders over the 8-week treatment.

**Results:**

The primary outcome which was percentage of the sustained CSBMs responders for the subset of women with severe constipation was significantly higher in the EA group (24.3%) than in the SA group (8.1%) with difference of 13.1% (95%CI, 6.5% to 19.7%; P<0.001). As for the secondary outcomes, responders for ≥9 of 12 weeks of follow-up were higher in the EA group than in the SA group. Additionally, EA had significantly better improvement in mean weekly CSBMs, mean weekly spontaneous bowel movements (SBMs), and mean score changes of stool consistency and straining as well as quality of life of patients. The incidence of adverse events (AEs) related to acupuncture was rare and no statistical significance was found between two groups.

**Conclusion:**

EA improved the spontaneity and the completeness of the bowel movement of women with severe functional constipation during 8-week treatment and the effect sustained for 12 weeks after stopping treatment.

## 1. Introduction

Chronic constipation is a common gastrointestinal disorder manifested with infrequent bowel movements, excessive straining, hard stools, and/or incomplete evacuation feelings [1]. The global prevalence of chronic idiopathic constipation was approximately 14% [2]. Various studies found that the prevalence of constipation in women is more than twice that of men of the same age [3, 4]. Women with constipation usually also have lower urinary tract symptoms of overactive bladder and urinary incontinence [5]. Women are more likely than men to have a poor quality of life [6] and seek medical help [1]. The mean annual total cost for healthcare was reported to be $7,522 for each patient with chronic constipation and women are more likely to use nonprescription and alternative medicine treatments [7]. Potential causes for this gender difference remain unknown; they may include physiological hormonal differences [8]. Hence, the purpose of this study was to explore efficacy of electroacupuncture (EA) on women with severe constipation.

Conventional treatments of chronic constipation include stimulant and osmotic medications, yet, due to concerns for efficacy and safety, nearly 50% of patients with chronic constipation were not satisfied with these pharmacological treatments [9]. Emerging evidence suggests that acupuncture may increase weekly spontaneous bowel movements (CSBMs), decrease constipation symptoms, and improve quality of life in patients with chronic constipation [10]. However, studies assessing whether female patients can benefit from acupuncture remain scarce [11].

The primary findings of the original multicenter randomized controlled trial on acupuncture for chronic severe functional constipation demonstrated that electroacupuncture increased mean weekly CSBMs during weeks 1 to 8 [12]. This study focused on the efficacy of acupuncture on subgroup of women with chronic severe functional constipation.

In order to extend the findings of the previous trial, a subgroup analysis of data was performed to specifically evaluate the efficacy and safety of electroacupuncture (EA) in women with chronic severe functional constipation.

## 2. Materials and Methods

### 2.1. Overview of the Original Trial

The acupuncture constipation study [12] was a multicenter, randomized, sham-acupuncture (SA) controlled study which was conducted at 15 hospitals in China. A total of 1075 patients with chronic severe functional constipation were recruited between 8 October 2012 and 4 May 2014 and were randomly assigned to EA and SA group. The study included 536 patients (415 female, 77.4%) in the EA group and 539 patients (407 female, 75.5%) in the SA group and 54 patients dropped out during study. After 2-week screening period, 8 weeks with 28 sessions of EA at traditional acupoints (bilateral ST25, SP14, and ST37) or SA at nonacupoints were performed. Participants were followed up for 12 weeks. Diagnosis was based on Rome III criteria for chronic functional constipation and severe constipation was defined as two or fewer CSBMs per week with hard stools, frequent straining, and the sensation of incomplete evacuation [14]. A CSBM was defined as a bowel movement with a sensation of complete evacuation that occurred without use of any medication or other methods to assist defecation in the previous 24 hours. This article presented the results of a secondary analysis that was not prespecified in the study protocol. The rationale, design, and methods of the acupuncture constipation study were presented in detail elsewhere [15].

The study protocol was performed in accordance with Declaration of Helsinki and approved by the ethics committees of each responsible site. The study protocol complied with Standards for Reporting Interventions in Clinical Trials of Acupuncture guidelines. The trial was registered at ClinicalTrials.gov (identifier NCT01726504).

### 2.2. Study Design

In this study, efficacy of EA on only female patients (76%) with chronic severe functional constipation was assessed. The European Medicines Agency (EMA) guideline [13] indicates that CSBMs for primary outcome should incorporate spontaneity, completeness of the bowel movement, and a responder analysis. Spontaneous bowel movements (SBMs) were defined as evacuation without manual methods or medication and complete spontaneous bowel movement (CSBM) was defined as SBM without sense of incomplete evacuation. According to this guideline, weekly responders were defined as patients achieving at least 3 CSBMs/week and, at the same time, an increase of at least 1 CSBM/week compared to baseline. Meanwhile, assessment of the primary outcome should be based on participants meeting the responder criteria for at least 75% of the duration of the treatment period (overall responders). In this analysis, “overall responders” were defined as responders for at least 6 of the 8 treatment weeks and “sustained responders” were defined as overall responders who also met the responder criteria for at least 3 of the last 4 treatment weeks. Participants were required to keep a stool diary every day for 22 weeks, from 2 weeks before randomization to the end of week 20 after randomization. The primary outcome of this subgroup analysis was the proportion of sustained responders during the 8-week treatment period. The secondary outcomes included the proportion of overall responders over weeks 1 to 8; the proportion of participants who met the responder criteria for at least 9 weeks of the 12 follow-up weeks; the proportion of weekly responders from weeks 1 to 20; change of weekly mean CSBMs from baseline during weeks 1-8 and 9-20; change of mean weekly SBMs during weeks 1-8; change from baseline of Bristol Stool Form Scale (BSFS) score and mean straining score based on SBMs during weeks 1-8; change from baseline of Patient Assessment of Constipation Quality of Life Questionnaire (PAC-QOL) score at weeks 4 and 8; the proportion of patients using other treatments and the mean weekly frequency of other treatments used during weeks 1-8 and 9-20.

### 2.3. Statistical Methods

The primary outcome was analyzed by fitting a generalized linear model for a binomial distribution, adjusted for baseline value and sites. The same approach was used for overall responders, participants who met the responder criteria for ≥ 9 of the 12 weeks of the follow-up period, and patients using other measures. The changes from baseline in mean CSBMs per week, mean SBMs per week, mean stool consistency, and mean straining scores and PAC-QOL score were assessed using a general linear model. Wilcoxon rank-sum test was used to compare the frequency of other treatments used per week. The proportions of weekly responders from weeks 1 to 20 were plotted and compared with a generalized linear model between groups.

Analyses were based on the intention-to-treat principle, with all randomly assigned participants included. Patients were considered nonresponders if they withdrew from study without providing data for CSBMs during the intervention or follow-up period. Descriptive statistics were used for demographics, baseline characteristics, and safety variables. All statistical analyses were performed using SAS version 9.4 (SAS Institute Inc.) with a 2-sided P value of less than .05 considered significant.

## 3. Results

### 3.1. Participant Characteristics

Details of participant flow through the acupuncture constipation study have been described elsewhere [12]. There were 822 female patients in this analysis, 415 in the EA group, and 407 in the sham-acupuncture (SA) group. At week 8, there were 47.39% (191/403) of female patients in the EA group and 79% (301/381) in the SA group which were treated as nonresponders with 12 and 26 lost to follow-up respectively. 780 (94.9%) completed the follow-up assessment. Baseline characteristics of patients were presented in Table 1.

### 3.2. Outcomes

**Figure 1:**
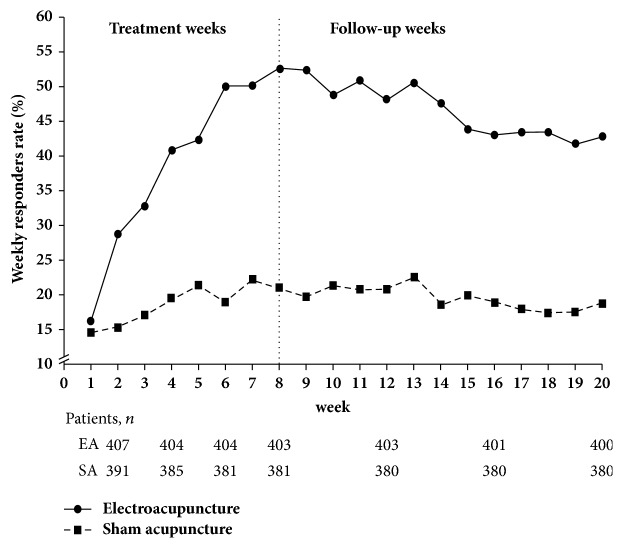
**Weekly responders rate in the electroacupuncture and the sham-acupuncture groups from week 1 to week 20. **Responders were defined as patients achieving at least 3 complete spontaneous bowel movements (CSBMs)/week and, at the same time, an increase of at least 1 CSBM/week compared to the baseline according to the guideline of European Medicines Agency [13]. 12 participants in the EA group and 26 in the SA group were treated as nonresponders. The differences in weekly responders between two groups were significant from week 2 till the end of the follow-up periods (P<0.01 for all) (Supplementary Table 1).

For the primary outcome, the proportion of sustained responders were statistically significantly higher in the EA group (24.3%) than the SA group (8.1%) with a difference of 13.1% (95%CI, 6.5% to 19.7%; P<0.001) (Table 2). For secondary outcomes, the percentage of overall responders was 24.8% in the EA group and 8.6% in the SA group with a difference of 12.5% (95%CI, 7.3% to 17.6%; P<0.001) (Table 2). The percentage of participants who met the responder criteria for more than 9 out of 12 weeks during the follow-up period in the EA group (38%) was substantially higher than that in the SA group (11.3%) with a difference of 23.9% (95%CI, 18.0% to 29.8%; P<0.001). The percentage of weekly responders was shown in Figure 1. There was no difference between the two groups at the end of week 1, but since week 2, the percentage of responders in the EA group had been consistently around twice higher than that in the SA group (P<0.001, for all) (Supplementary Table 1). At week 8, the percentage of responders in the EA group reached the highest which was 51.1% compared to 19.7% in the SA group.

The increases in the mean CSBMs per week from baseline to weeks 1-8 and weeks 9-20 were significantly higher in the EA group than the SA group with between-group differences of 0.9 (95%CI, 0.5 to 1.3; P<0.001) and 1.1 (95%CI, 0.7 to 1.5; P <0.001) during the treatment and follow-up periods, respectively. The EA group also showed significantly greater increases in mean weekly SBMs than the SA group during weeks 1-8 with a between-group difference of 1.0 (95%CI, 0.8 to 1.2; P<0.001). Mean score change of stool consistency and straining from baseline were statistically better in the EA group than the SA group during weeks 1-8 (P <0.001 for both) with between-group differences of 0.2 (95%CI, 0.1 to 0.3; P<0.001) and -0.2 (95%CI, -0.3 to -0.2; P<0.001), respectively.

The improvements of PAC-QOL overall score were significantly greater in the EA group than the SA group with between-group differences of -0.2 (95%CI, -0.2 to -0.1; P<0.001) and -0.3 (95%CI, -0.4 to -0.2; P<0.001) at week 4 and week 8, respectively. No differences were found between groups in the proportion or frequency of patients using other treatments during weeks 1-8 or weeks 9-20 (P > 0.05 for all).

### 3.3. Adverse Events

Throughout the trial, 16 participants (3.9%) in the EA group and 11 participants (2.7%) in the SA group had adverse events (AEs) related to acupuncture with local hematoma as the most common in both groups (Table 3). No difference was found between groups in the proportions of participants with acupuncture-related AEs (p > 0.05). AEs unrelated to treatment were rare (Supplementary Table 2).

## 4. Discussion

This study demonstrated that 8 weeks' EA treatment has many positive effects for relieving symptoms in women with chronic severe functional constipation as evidenced by a higher proportion of sustained responders (24.3% in the EA group versus 8.1% in the SA group) in regaining CSBMs. Moreover, the proportion of weekly responders for more than 9 weeks of 12 weeks' follow-up period was 38.1% in the EA group and 11.3% in the SA group with the difference of 23.9% between two groups. EA may thus be an effective alternative for the management of chronic constipation in women and the effectiveness could last for 12 weeks without treatment.

To the best of our knowledge, this is the first study assessing the effectiveness of acupuncture for women with chronic constipation. Responder analyses were frequently used in pharmaceutical trials for chronic constipation. In the study including 1346 patients (80.8% female patients), the percentages of sustained responders were 21% and 19.5% after 12 weeks of 3mg and 6mg plecanatide treatment as compared to 10.2% in the group treated with placebo [16]. In the study with 483 patients (91.5% female), 15.7% patients met the criteria as overall responders after 12-week once-daily linaclotide 145 *μ*g [17]. Then, in a follow-up trial of 1223 patients (78.8% female patients), the percentages of overall and sustained responders were found to be 13.4% and 12.4% with 12-week once-daily linaclotide 72 *μ*g and 12.4% and 11.2% with 12-week once-daily linaclotide 145 *μ*g [18]. In this analysis, after 8 weeks' EA treatment, the percentage of overall responders was 24.8% and the percentage of sustained responders was 24.3%. These rates appear higher than the studies of plecanatide and linaclotide [16–18], even though direct comparison between the present study and previous pharmaceutical trials is inappropriate.

An integrated analysis of 4 double-blind, placebo-controlled, phase III studies of Asian and non-Asian women showed that after 2 mg prucalopride at week 12, the percentage of responders who have at least 3 CSBMs a week was approximately 25% to 34% [17]. Therapeutic gain over placebo in 3 of these 4 studies is clinically relevant [17]. In our study, the responders at week 8 were 52.6% and 21.0% in the EA group and SA group.

Additionally, in this analysis, EA was also found effective in increasing weekly CSBMs and improving quality of life in female patients with chronic severe functional constipation. The change of mean weekly CSBMs from baseline in the EA group was 1.7 during weeks of 1-8 and 1.9 during weeks of 9-20 which was consistent with the results in the original trial (1.76 and 1.96). The consistency of results may show the stability of acupuncture effect. In previous pharmaceutical trials, the change from baseline in mean weekly CSBMs after 12-week treatment of prucalopride 2mg ranged from 1.6 to 2.6 [9, 14, 19, 20]. The benefits of prucalopride are limited by the lack of follow-up periods in all these previous trials [9, 14, 19, 20], whereas results of this analysis indicate that EA effects could last for 12 weeks after 8 weeks of treatment.

The change of overall PAC-QOL score in the present study was 0.9 and 0.6 in the EA and the SA groups, respectively. A multicenter, randomized, placebo-controlled trial [21] evaluated the efficacy of 2mg and 4 mg prucalopride on the quality of life of 713 (90.8% female) patients with severe chronic constipation who were dissatisfied with laxatives. The change of overall score of PAC-QOL at week 12 was 0.38, 0.65, and 0.66 in 2mg, 4mg, and placebo group, respectively. The analyses of other secondary outcomes including SBMs, stool consistency, and straining as well as side effects support the effectiveness and safety of EA in women with chronic constipation.

Acupuncture effects may result from both biological and psychological influences of acupuncture on the human body [22]. EA could have a multitude of physiological effects, but patient expectation also plays an important role in the effectiveness of acupuncture [23, 24].

The decision for patients to accept and healthcare providers to recommend a treatment usually depends on not only the effectiveness of the treatment but also economic costs. Although the total cost for 28 sessions ($80 USD each session) of EA seems far more expensive than pharmaceutical interventions [25], the long-lasting benefits as evidenced by the persistent improvements of constipation symptoms during the 12 weeks follow-up without treatment may indicate EA treatment is cost-effective. Nonetheless, cost effectiveness studies are warranted in future acupuncture trials to test this hypothesis. These understandings may shed light on the reasons why women are more likely to use nonprescription and alternative medicine treatments for constipation.

## 5. Limitations

This subgroup analysis has some limitations. First, only Chinese female patients were included in this secondary analysis which may limit the generalizability of the findings to other countries. Second, the subgroup analysis was not prespecified in the original trial.

## 6. Conclusions

8-week treatment of EA significantly improved the spontaneity and the completeness of the bowel movement in women with severe functional constipation and the effect could sustain for 12 weeks after stopping treatment. EA is safe and could be a promising alternative for the treatment of women with chronic severe functional constipation.

## Figures and Tables

**Table 1:** Baseline characteristics of the randomized participants.

**Characteristics**	**EA (n=415)**	**SA (n=407)**
Age, mean (SD), y	45.60 (15.69)	45.49 (15.11)
Participants with age <65 y	371 (89.40)	369 (90.66)
Participants with age ≥65 y	44 (10.60)	38 (9.34)
Race		
Han	401 (96.63)	396 (97.30)
Minorities	14 (3.37)	11(2.70)
BMI, mean (SD), kg/m^2^	22.12 (3.04)	22.49 (3.11)
Constipation duration, mean (SD), mo.	135.80 (122.26)	145.00 (130.29)
Coexisting illness	83 (20.00)	77 (18.92)
Others	55 (13.25)	56 (13.76)
Hypertension	28 (6.75)	21 (5.16)
Digestive system diseases	6 (1.45)	10 (2.46)
Diabetes mellitus	8 (1.93)	3 (0.74)
CSBMs/week, mean (SD)	0.40 (0.63)	0.40 (0.62)
SBMs/week, mean (SD)	1.89 (1.32)	2.03 (1.41)
Mean stool consistency of SBMs, mean (SD)^*∗*^	2.58 (1.13)	2.59 (1.10)
Mean straining of SBMs, mean (SD)^*∗*^	1.65 (0.66)	1.69 (0.61)
PAC-QOL score, mean (SD)	2.75 (0.69)	2.69 (0.69)
Patients using other treatments		
Rescue medicine	126 (30.36%)	103 (25.30%)
Others	14 (3.37%)	13 (3.19%)
Weekly frequency of other treatments used, median (IQR) ^†^		
Rescue medicine	1.25 (0.50-2.00)	1.00 (1.00-2.00)
Others	1.25 (0.50-4.00)	1.00 (0.50-3.00)

Data are n (%) unless otherwise stated.

Abbreviations: EA, electro-acupuncture; SA, sham acupuncture; CSBMs, complete spontaneous bowel movements; SBMs, spontaneous bowel movements; BMI, body mass index; PAC-QOL, Patient Assessment of Constipation Quality of Life Questionnaire; SD, standard deviation; IQR, interquartile range. Coexisting illness with different types occurring in a single participant were defined as independent coexisting illness.

^*∗*^Mean stool consistency and straining were evaluated based on the stools from spontaneous bowel movements. We could not obtain data from participants who had no SBMs during the baseline period. These two outcomes were both missing on 37 participants in the EA group and 35 in the SA group at baseline. Therefore, data for these two outcomes were from 378 participants in the EA group and 372 in the SA group.

^†^ The weekly frequency of other treatments was assessed in the patients who used other treatments for constipation.

**Table 2:** Outcome measures of intention-to-treat population.

**Outcomes**	**EA group**	**SA group**	**Between-group Difference**	**P Value**
	**(n=415)**	**(n=407)**	**Value (95%CI)**	
**Primary Outcome**				
Sustained responders (%) ^*∗*^	101 (24.34)	33 (8.11)	13.10% (6.50% - 19.70%)	<0.001
**Secondary Outcomes**				
Overall responders (%) ^†^	103 (24.82)	35 (8.60)	12.45% (7.26% -17.64%)	<0.001
Responders at week 8 (%) ^‡^	212 (51.08)	80 (19.65)	28.31% (21.86% - 34.73%)	<0.001
Responders for ≥ 9 of 12 weeks of follow-up weeks ^§^	158 (38.07)	46 (11.30)	23.90% (18.02% - 29.78%)	<0.001
Change in mean CSBMs/week from baseline, mean (95%CI)				
Weeks 1-8	1.73 (1.40 - 2.06)	0.80 (0.47 - 1.13)	0.93 (0.52 - 1.33)	<0.001
Weeks 9-20	1.92 (1.59 - 2.25)	0.80 (0.47 - 1.13)	1.13 (0.73 - 1.53)	<0.001
Change in mean SBMs/week during weeks 1-8, mean (95% CI)^*‖*^	2.29 (2.13 - 2.45)	1.31 (1.15 - 1.47)	0.98 (0.75 - 1.20)	<0.001
Change in stool consistency of SBMs during weeks 1-8, mean (95% CI)^*‖*^	0.85 (0.77 - 0.93)	0.64 (0.56 - 0.71)	0.21 (0.11 - 0.33)	<0.001
Change in straining of SBMs during weeks 1-8 mean (95% CI)^*‖*^	-0.71 (-0.76 to -0.66)	-0.48 (-0.53 to -0.43)	-0.22 (-0.29 to -0.16)	<0.001
Change in PAC-QOL score, mean (95% CI)				
Week 4	-0.50 (-0.54 to -0.45)	-0.35 (-0.40 to -0.30)	-0.15 (-0.21 to -0.08)	<0.001
Week 8	-0.87 (-0.93 to -0.82)	-0.56 (-0.62 to -0.51)	-0.31 (-0.38 to -0.24)	<0.001
Patients using other treatments				
Weeks 1-8				
Rescue medicine	121 (29.20)	137 (33.70)	-5.50% (-11.90% - 0.90%)	0.09
Others	12 (2.90)	12 (3.00)	0.80% (-6.90% - 8.50%)	0.83
Weeks 9-20				
Rescue medicine	77 (18.60%)	88 (21.60%)	-3.3% (-10.20% - 3.60%)	0.35
Others	9 (2.20%)	12 (3.00%)	-0.4% (-8.70% - 8.00%)	0.93
Weekly frequency of other treatments used, median (IQR) ^¶^				
Weeks 1-8				
Rescue medicine	0.63 (0.25 - 1.25)	0.75 (0.38 - 1.50)	NA	0.12
Others	0.44 (0.19 - 1.00)	0.19 (0.13 - 0.69)	NA	0.23
Weeks 9-20				
Rescue medicine	0.75 (0.33 - 1.42)	0.75 (0.33 - 1.67)	NA	0.50
Others	0.25 (0.17 - 0.42)	0.42 (0.83 - 1.63)	NA	0.83

Data are n (%) unless otherwise stated.

Abbreviations: EA, electro-acupuncture; SA, sham acupuncture; CSBMs, complete spontaneous bowel movements; SBMs, spontaneous bowel movements; BMI, body mass index; PAC-QOL, Patient Assessment of Constipation Quality of Life questionnaire; SD, standard deviation; IQR, interquartile range, NA, not applicable.

^*∗*^ Sustained responders were defined as responders for treatment for ≥ 6 of 8 weeks, including 3 of 4 final treatment weeks according to the guideline of European Medicines Agency [13], 12 participants in the EA group and 26 in the SA group were treated as non-responders.

^†^ Overall responders were defined as responders for treatment for ≥ 6 of 8 treatment weeks according to the guideline of European Medicines Agency [13], 12 participants in the EA group and 26 in the SA group were treated as non-responders.

^‡^ Responders were defined as patients achieving at least 3 CSBMs/week and, at the same time, an increase of at least 1 CSBM/week compared to baseline according to the guideline of European Medicines Agency [13], 12 participants in the EA group and 26 in the SA group were treated as non-responders.

^§^ 15 participants in the EA group and 27 in the SA group were treated as non-responders.

*‖* Stool consistency and straining were evaluated based on the stools from spontaneous bowel movements. We could not obtain data from participants who had no SBMs during the baseline and/or the treatment period. These two outcomes were both missing on 37 participants in the EA group and 35 in the SA group at baseline. Therefore, data for these two outcomes were from 378 participants in the EA group and 372 in the SA group.

¶The weekly frequency of other treatments used was assessed in the patients who used other treatments for constipation.

**Table 3:** Adverse events related to treatment.

	**EA (** **n** = 414^†^**)**	**SA (** **n** = 406^†^**)**
Total adverse events	23 (5.55)	16 (3.94)
Hematoma around the site of needling	13 (3.14)	11 (2.71)
Severe sharp pain (VAS ≥ 7)	2 (0.48)	4 (0.98)
Sharp pain lasting more than 0.5 hour	2 (0.48)	1 (0.25)
Fever	1 (0.25)	0 (0.00)
Fever with abdominal pain	1 (0.25)	0 (0.00)
Fatigue	1 (0.25)	0 (0.00)
Insomnia	1 (0.25)	0 (0.00)
Other discomforts	3 (0.72)	0 (0.00)

Adverse events were analyzed in all participants who received treatment. Adverse events were counted by type rather than frequency in the same participant. Adverse events with different types occurring in a single participant were defined as independent adverse events. An adverse event with multiple occurrences in a single participant was defined as 1 adverse event.

^†^ Data on safety evaluation for 2 participants were not available (1 in EA group and 1 in SA group).

## Data Availability

The data used to support the findings of this study are included within the article.

## Abbreviations

EA: Electroacupuncture
CMBMs: Complete spontaneous bowel movements
EMA: European Medicines Agency
SBMs: Spontaneous bowel movements
BSFS: Bristol Stool Form Scale
PAC-QOL: Patient Assessment of Constipation Quality of Life Questionnaire
SA: Sham-acupuncture
AEs: Adverse events.

## Supplementary Materials

Supplementary Materials Supplementary Table 1. The difference in weekly responders between groups Supplementary Table 2. Adverse events unrelated to treatment Supplementary File. CONSORT checklist Supplementary File. Ethics committee approval.
