# Supplementary material for: Electroacupuncture for Women with Chronic Severe Functional Constipation: Subgroup Analysis of a Randomized Controlled Trial
Source: Biomed Res Int. 2019 Jan 13;2019:7491281. doi: 10.1155/2019/7491281 (PMC6348848; doi:10.1155/2019/7491281)
Supplement: Supplementary Materials — Supplementary Table 1. The difference in weekly responders between groups Supplementary Table 2. Adverse events unrelated to treatment Supplementary File. CONSORT checklist Supplementary File. Ethics committee approval. [file 7491281.f1.zip › mat.7491281.v2 (2).pdf]

Supplementary Table 1. The difference in weekly responders between groups

	EA group (N = 415)	SA group (N = 407)	Total (N = 822)	*P* value *
**Treatment period**				
Week 1				
Non-responder	342 (83.8)	338 (85.4)	680 (84.6)	0.~~548~~7599~
Responder	66 (16.2)	58 (14.6)	124 (15.4)	
Week 2				
Non-responder	290 (71.3)	331 (84.7)	621 (77.8)	<0.0002~~1~~~
Responder	117 (28.7)	60 (15.3)	177 (22.2)	
Week 3				
Non-responder	272 (67.2)	320 (82.9)	592 (74.8)	<0.001~
Responder	133 (32.8)	66 (17.1)	199 (25.2)	
Week 4				
Non-responder	239 (59.2)	310 (80.5)	549 (69.6)	<0.001~
Responder	165 (40.8)	75 (19.5)	240 (30.4)	
Week 5				
Non-responder	233 (57.7)	301 (78.6)	534 (67.9)	<0.001~
Responder	171 (42.3)	82 (21.4)	253 (32.1)	
Week 6				
Non-responder	202 (50.0)	309 (81.1)	511 (65.1)	<0.001~
Responder	202 (50.0)	72 (18.9)	274 (34.9)	
Week 7				
Non-responder	201 (49.9)	296 (77.7)	497 (63.4)	<0.001~
Responder	202 (50.1)	85 (22.3)	287 (36.6)	
Week 8				
Non-responder	191 (47.4)	301 (79.0)	492 (62.8)	<0.001~
Responder	212 (52.6)	80 (21.0)	292 (37.2)	
**Follow-up period**				
Week 9				
Non-responder	192 (47.6)	305 (80.3)	497 (63.5)	<0.001~
Responder	211 (52.4)	75 (19.7)	286 (36.5)	
Week 10				
Non-responder	206 (51.1)	299 (78.7)	505 (64.5)	<0.001~

	EA group (N = 415)	SA group (N = 407)	Total (N = 822)	*P* value [*]
Responder	197 (48.9)	81 (21.3)	278 (35.5)	
Week 11				
Non-responder	198 (49.1)	301 (79.2)	499 (63.7)	<0.001~
Responder	205 (50.9)	79 (20.8)	284 (36.3)	
Week 12				
Non-responder	209 (51.9)	301 (79.2)	510 (65.1)	<0.001~
Responder	194 (48.1)	79 (20.8)	273 (34.9)	
Week 13				
Non-responder	198 (49.4)	294 (77.4)	492 (63.0)	<0.001~
Responder	203 (50.6)	86 (22.6)	289 (37.0)	
Week 14				
Non-responder	210 (52.4)	309 (81.3)	519 (66.5)	<0.001~
Responder	191 (47.6)	71 (18.7)	262 (33.5)	
Week 15				
Non-responder	225 (56.1)	304 (80.0)	529 (67.7)	<0.001~
Responder	176 (43.9)	76 (20.0)	252 (32.3)	
Week 16				
Non-responder	228 (56.9)	308 (81.1)	536 (68.6)	<0.001~
Responder	173 (43.1)	72 (18.9)	245 (31.4)	
Week 17				
Non-responder	227 (56.6)	312 (82.1)	539 (69.0)	<0.001~
Responder	174 (43.4)	68 (17.9)	242 (31.0)	
Week 18				
Non-responder	226 (56.5)	314 (82.6)	540 (69.2)	<0.001~
Responder	174 (43.5)	66 (17.4)	240 (30.8)	
Week 19				
Non-responder	233 (58.3)	313 (82.4)	546 (70.0)	<0.001~
Responder	167 (41.8)	67 (17.6)	234 (30.0)	
Week 20				
Non-responder	229 (57.3)	309 (81.3)	538 (69.0)	<0.001~
Responder	171 (42.8)	71 (18.7)	242 (31.0)	

Abbreviations: EA, electro-acupuncture; SA, sham acupuncture.

Weekly responders were defined as patients achieving at least 3 complete spontaneous bowel movements (CSBMs)/week and, at the same time, an increase of at least 1 CSBM/week compared to baseline according to the guideline of European Medicines Agency [16]. 12 participants in the EA group and 26 in the SA group were treated as non-responders.

* Calculated using the generalized linear model with a binomial distribution, adjusted for baseline value and sites.~~Fisher exact test~~.

Supplementary Table 2. Adverse events unrelated to treatment

	**EA (n=414 *)**	**SA (n=406 *)**
Total adverse events	16 (3.9)	11 (2.7)
Common cold	6 (1.4)	4 (1.0)
Upper respiratory infection	3 (0.7)	0 (0.0)
Fever	1 (0.2)	0 (0.0)
Fever with abdominal pain	1 (0.2)	0 (0.0)
Pneumonia	0 (0.0)	1 (0.2)
Abdominal pain	1 (0.2)	0 (0.0)
Calcium deficiency	1 (0.2)	0 (0.0)
Cough	0 (0.0)	0 (0.0)
Stomach pain	0 (0.0)	1 (0.2)
Palpitation	0 (0.0)	1 (0.2)
Discomfort at the chest area	0 (0.0)	1 (0.2)
Hyperglycemia	1 (0.2)	0 (0.0)
Acne	0 (0.0)	1 (0.2)
Lumbar sprain	1 (0.2)	0 (0.0)
Body pain after 2 weeks of treatment	0 (0.0)	1 (0.2)
Sacroiliitis	1 (0.2)	0 (0.0)

Abbreviations: EA = electro-acupuncture; SA = sham acupuncture.

Adverse events were analyzed in all participants who received treatment. Adverse events were counted by type rather than frequency in the same participant. Adverse events with different types occurring in a single participant were defined as independent adverse events. An adverse event with multiple occurrences in a single participant was defined as 1 adverse event.

* Data on safety evaluation for 2 participants were not available (1 in EA group and 1 in SA group).
